# Commonly Used Severity Scores Are Not Good Predictors of Mortality in Sepsis from Severe Leptospirosis: A Series of Ten Patients

**DOI:** 10.1155/2012/532376

**Published:** 2012-05-21

**Authors:** Dimitrios Velissaris, Menelaos Karanikolas, Nikolaos Flaris, Fotini Fligou, Markos Marangos, Kriton S. Filos

**Affiliations:** ^1^Department of Anesthesiology and Critical Care Medicine, Patras University Hospital, 26500 Rion, Greece; ^2^Department of Anesthesiology, Washington University School of Medicine, Campus Box 8054, 660 S. Euclid Avenue, St. Louis, MO 63110, USA; ^3^Department of Anesthesiology and Critical Care Medicine, Patras University School of Medicine, 26500 Rion, Greece; ^4^Department of Internal Medicine, Patras University School of Medicine, 26500 Rion, Greece

## Abstract

*Introduction*. Severe leptospirosis, also known as Weil's disease, can cause multiorgan failure with high mortality. Scoring systems for disease severity have not been validated for leptospirosis, and there is no documented method to predict mortality. *Methods*. This is a case series on 10 patients admitted to ICU for multiorgan failure from severe leptospirosis. Data were collected retrospectively, with approval from the Institution Ethics Committee. *Results*. Ten patients with severe leptospirosis were admitted in the Patras University Hospital ICU in a four-year period. Although, based on SOFA scores, predicted mortality was over 80%, seven of 10 patients survived and were discharged from the hospital in good condition. There was no association between SAPS II or SOFA scores and mortality, but survivors had significantly lower APACHE II scores compared to nonsurvivors. *Conclusion*. Commonly used severity scores do not seem to be useful in predicting mortality in severe leptospirosis. Early ICU admission and resuscitation based on a goal-directed therapy protocol are recommended and may reduce mortality. However, this study is limited by retrospective data collection and small sample size. Data from large prospective studies are needed to validate our findings.

## 1. Introduction

Sepsis and multiple organ failure are associated with high ICU morbidity and mortality. Currently available organ failure scoring systems, such as the Sequential Organ Failure Assessment (SOFA) score, can help assess organ dysfunction over time and have been associated with ICU mortality.

The term leptospirosis refers to disease caused by any leptospira, regardless of specific serotype. Leptospirosis is thought to be the most widespread zoonosis in the world. Weil syndrome [1, 2] is defined as severe leptospirosis with multiorgan dysfunction. Weil syndrome can cause respiratory failure [3], renal failure requiring hemodialysis [4], hemorrhages, anemia, disturbances in consciousness, and persistent fever and sepsis with high mortality, even in previously healthy patients [5].

SOFA score has been shown to correlate with outcome in a variety of ICU populations [6]. Therefore, use of repeated SOFA score measures over time is a reasonable attempt to assess the severity of organ dysfunction and predict outcome in severe leptospirosis, even though SOFA score has not been validated in patients with leptospirosis. However, our experience with patients admitted to the ICU for severe leptospirosis suggests that patients can have good outcome despite severe multiorgan failure with high SOFA scores and very high predicted mortality on ICU admission. This retrospective study was conducted in an attempt to evaluate whether admission SOFA scores, repeated SOFA score measurements over time, admission SAPS II or admission APACHE II scores can help predict mortality in severe leptospirosis.

## 2. Materials and Methods

### 2.1. Study Design and Data Collection

This retrospective study consisted of chart review and data extraction from all confirmed leptospirosis cases admitted to our ICU over a 4-year period. The project was approved by the Institution Ethics Committee. Demographic data and data on clinical history, physical examination at the time of ICU admission, initial and follow-up laboratory values, diagnostic tests used to confirm leptospirosis, and treatment provided were collected from each medical record and stored without any personally identifiable information in a secure electronic database. SOFA scores on admission and every day until death or discharge from the ICU were also recorded. Calculation of daily SOFA scores was based on the most abnormal value for each SOFA score parameter each day [7]. APACHE II and SAPS II were also calculated on the day of ICU admission. When patients were sedated and intubated, we assumed they did not have any CNS abnormalities.

### 2.2. Statistical Analysis

Because data distribution was skewed and violated normality, comparisons between survivors and nonsurvivors were done with the nonparametric Mann-Whitney *U* test. Observed mortality was compared with expected mortality using Fisher's exact test. Data analysis was done with the SPSS version 17.0 statistical software package (SPSS Inc, Chicago, IL, USA). *P* < 0.05 was considered significant for all comparisons. Fisher's exact test was done using the STATCALC component of the Epi Info statistics software package (freely available from the Centers for Disease Control at http://www.cdc.gov/Epiinfo/, January 5, 2010).

## 3. Results

### 3.1. Demographic and Clinical Data

Ten patients (9 men, one woman) were admitted to the ICU for multiorgan failure due to severe leptospirosis between 2006 and 2009. All patients lived and worked in rural areas and therefore were at risk for occupational exposure to leptospira. Because data did not have a normal distribution, data in Table 1 are presented as median (minimum, maximum).

### 3.2. Initial Diagnosis and ICU Therapy

All patients in this case series were admitted to the ICU because of severe respiratory insufficiency, acute renal failure, hypotension, coagulopathy, coma, or any combination of the above. All patients presented in the Emergency Department with a GCS of 15, except a female patient who had a GCS of 6, intubated initially in the ED and had the shortest ICU length of stay (died after 4 days). None of the patients developed lung hemorrhages or cardiac rhythm abnormalities either on admission or during ICU stay. Demographic and clinical data for each patient are presented in Table 2.

At the time of ICU admission, the diagnosis of leptospirosis was suspected on the basis of history and clinical presentation. Diagnosis was later confirmed on all patients by enzyme-linked immunosorbent assay (ELISA) [8]. Treatment in all 10 cases included broad spectrum antibiotics, oxygen supplementation by face mask, CPAP or intubation and mechanical ventilation, and support of all failing systems. Hemodynamic resuscitation and stabilization were based on the goal-directed therapy protocol described by Rivers et al. [9], in an attempt to optimize vital organ perfusion and oxygen delivery to peripheral tissues.

### 3.3. Disease Severity Scores on ICU Admission

All nine men were fully awake and oriented on ICU admission (GCS = 15), whereas the only female patient was comatose (GCS = 6), without evidence of meningeal infection on CSF testing.

On ICU admission, oxygenation impairment was significantly worse in nonsurvivors (*n* = 3) compared to survivors (*n* = 7): Median PaO_2_/FiO_2_ was 105 in nonsurvivors versus 215 in survivors (*P* = 0.04). Similarly, admission platelet count was significantly lower in nonsurvivors (median 21,000) versus survivors (median 43,000, *P* = 0.05).

The cause of death in all three nonsurvivors was multiple organ failure and increased need for inotropic and vasoactive agents as shown in Table 2. No arrhythmias were recorded during their ICU stay.

SOFA, APACHE II, and SAPS II on ICU admission are presented in Table 3. With regards to mortality, although the predicted mortality based on initial SOFA score was high, only 3 of 10 patients died.

## 4. Discussion

### 4.1. Leptospirosis in the ICU

Leptospirosis is a worldwide zoonosis [10, 11] and has been reported in most parts of the world, including North America (USA [8, 12, 13]), Europe (Germany [14], The Netherlands [15], Portugal [16, 17], Greece [18], Serbia [19], Turkey [20, 21]), The Middle East (Israel [22, 23]), Central and South America (Brazil [4, 24, 25], Cuba [26], Nicaragua [27], Trinidad and Tobago [28], West Indies [29]), Asia (India [30, 31], Thailand [32], Indonesia [3]), Africa (Egypt [33, 34], Nigeria [35, 36], South Africa [37]), Australia [38], and New Zealand [39, 40]. Leptospirosis is characterized by great clinical variability, ranging from a mild flu-like illness to life-threatening multiorgan failure. There are no established criteria for early identification of the severe forms of leptospirosis but prompt recognition of severe cases followed by appropriate timely intervention may contribute to improved morbidity and mortality. Several studies attempted to identify prognostic factors associated with mortality, but different authors have reached different conclusions. Dupont demonstrated that dyspnea, oliguria, low white blood cell count, repolarization abnormalities on electrocardiogram, and alveolar infiltrates on chest radiographs were independently associated with mortality [29], whereas Esen et al. [20] demonstrated that patients with altered mental status and hyperkalemia on hospital admission are at high risk for mortality and should be followed up in an ICU. A prospective cohort study by Panaphut et al. [32] suggested that oliguria, hyperkalemia, pulmonary rales, or hypotension on admission are associated with high mortality, a study by Marotto et al. [25] identified hemodynamic disturbance, renal dysfunction, and hyperkalemia as variables associated with mortality. In addition, a large recent study from India by Pappachan et al. [41] used logistic regression and identified pulmonary and central nervous system involvement as significant predictors of death, whereas a large recent study from Indonesia [3] identified pulmonary involvement as a strong independent predictor of mortality. Despite differences between studies, most authors agree that intensive care and early intervention should be provided for patients who present with risk factors and in cases where there is high index of clinical suspicion for leptospirosis, but diagnosis has not been confirmed by the laboratory.

 Mortality in leptospirosis has been reported to be up to 55% in different studies but varies depending on case mix and severity of organ dysfunction. In a study from Brazil by Vieira and Brauner [42], where all 35 patients presented with ARF required mechanical ventilation and developed multiorgan failure, mortality was 51%. [42]. In another study from India, all 60 leptospirosis patients who were admitted to the ICU had evidence of severe sepsis, 46 of 60 had multiple organ dysfunction, 26 of 60 required ventilatory support, and mortality was 52% [30]. In contrast, although only patients with severe leptospirosis were admitted to the ICU in our study, mortality was lower (3 of 10 patients, 30%) compared to previous reports.

### 4.2. SOFA Score and Other Severity Scoring Systems

Currently available outcome prediction models include the APACHE II (Acute Physiology and Chronic Health Evaluation II), SAPS II (Simplified Acute Physiology Score II), and MPM (Mortality Probability Models). These systems calculate a prediction based on values recorded within the first 24 hours after ICU admission. The APACHE-II score estimates ICU mortality based on clinical signs and laboratory values but also takes into account both acute and chronic patient diseases. The SAPS II is a severity of disease classification system, and it is mostly used to describe morbidity and outcome. Because of the need to evaluate changes in patient status over time, two scoring systems were developed, the Multiple Organ Dysfunction Score (MODS) and the Sequential Organ Failure Assessment (SOFA) scores [7, 43], which are calculated on admission and every 24 hours until patient death or discharge from the ICU.

The MODS was developed based on a literature review of all studies related to multiple organ dysfunction published between 1969 and 1993, to determine which characteristics had been used to define organ failure. The SOFA score was created during a consensus conference organized by the European Society of Intensive Care and Emergency Medicine. Both scores calculate a summary value for the degree of dysfunction for six organs (respiratory, hematologic, cardiovascular, liver, renal, and central nervous system). For SOFA score, this evaluation includes PO_2_/FiO_2_ ratio, levels of serum creatinine, levels of serum bilirubin, platelet count, assessment of neurologic status (Glasgow Coma Scale) and assessment of the cardiovascular system (doses of adrenergic agents administered for hypotension). The main difference between the MODS and the SOFA is in the evaluation of cardiovascular function. In MODS, the cardiovascular assessment is based on the pressure-adjusted heart-rate (PAR), defined as the product of the heart rate (HR) multiplied by the ratio of the right atrial pressure (RAP) to the mean arterial pressure (MAP), whereas the SOFA score is calculated based on mean arterial pressure and the need for vasopressors.

Results from many clinical studies show that the MODS and the SOFA score correlate well with outcome in terms of mortality [44] and also correlate with the APACHE II score. Of note, compared to the APACHE II score, both MODS and SOFA scores were better predictors of mortality in the subgroup of patients with shock. With regards to the SOFA score, although this was not created for use as predictor of mortality, SOFA score changes over time have been associated with ICU mortality. In each 24-hour period, the most abnormal value for each parameter was used in the calculation of the SOFA score [7].

### 4.3. Leptospirosis in the ICU: Our Results

The findings of our study come in line with the results of a retrospective study by Ittyachen [31] on 104 cases with clinical suspicion of severe leptospirosis. In this study, leptospirosis was serologically confirmed in 53 (50.7%) cases, and mortality was 26.8% in the seronegative group versus 3.8% in the seropositive group. The discordance between observed mortality and SOFA-predicted mortality in our study could be attributed to statistical error due to our small number of patients or errors in calculating mortality predicted by SOFA score. Other plausible explanations include real differences between our leptospirosis ICU patient population and the SOFA reference population [43] or greater potential for organ recovery when organ dysfunction is caused by leptospirosis. Admission SOFA scores were high in both survivors and nonsurvivors. SOFA scores declined during the first 5 ICU days due to restoration of platelet count and hemodynamic stabilization in all patients. However, improvement was more pronounced in survivors. Over time, SOFA scores continued to improve, due to gradual restoration of respiratory, renal and hepatic function in survivors, whereas SOFA scores remained high until death in nonsurvivors. However, the results of this small, retrospective study should be interpreted with caution, because the retrospective nature of our study and the small sample size are major shortcomings of this work.

## 5. Conclusion

Leptospirosis is a severe disease that can progress to multiorgan failure requiring ICU care, with high predicted mortality. Although SOFA score can be used as a tool to assess disease severity in leptospirosis, our limited data suggest that SOFA and other commonly used severity scores may not be good predictors of mortality. Our findings suggest that we should be optimistic in cases of severe leptospirosis with multiple organ failure, as in this series critically ill leptospirosis patients with high disease severity scores had a good outcome. Prospective multicenter studies with large numbers of patients are needed to validate our findings.

## Figures and Tables

**Table 1:** Demographic and Clinical Data on ICU admission in survivors and nonsurvivors.

Variable	Overall	Survivors	Nonsurvivors
Age	54 (22, 74)	56 (22, 74)	52 (50, 58)
ICU LOS	10.5 (4,37)	17 (5, 37)	8 (4, 13)
PaO_2_/FiO_2_	155 (70, 338)	215 (142, 338)	105 (70, 150)
Platelet count	35 (15, 82)	43 (20, 82)	21 (15, 28)
Bilirubin	8.64 (1, 17)	9.33 (1, 14)	7 (2, 17)
Creatinine	2.9 (1, 6)	3.7 (1, 6)	3 (3, 4)
Ventilator days	6 (0, 27)	0 (0, 27)	8 (4, 13)
SOFA score	15.5 (9, 18)	14 (9, 18)	17 (17, 18)

PaO_2_/FiO_2, _with PaO_2_ measured in mmHg, Platelet count expressed in thousands/mm^3^, Bilirubin measured as mg/dL, Creatinine measured as mg/dL.

Values are presented as median (minimum, maximum).

**Table 2:** Demographic and clinical data for each patient.

Case no.	Age	ICU LOS	Initial GCS	PaO_2_/FiO_2_	Inotrope/vasopressor days	Serum Cr	Bili	PLT *10^3^	SOFA/predicted mortality	APACHE II/predicted mortality	SAPS II/predicted mortality	Outcome
1	50	8	15	105	D8	2.9	16.9	21	17/95	16/25	50/50	Death
2	56	32	15	160	N25	5.4	10.02	20	18/95	17/25	68/75	Good
3	52	37	15	150	D12	2.9	7.08	50	14/95	16/25	65/75	Good
4	22	17	15	142	D6	6.1	10.6	30	17/95	13/15	50/50	Good
5	58	4	6	150	N40	2.5	1.52	28	17/95	18/25	34/15	Death
6	67	6	15	215	D3	2.7	1.23	82	9/33	4/4	22/5	Good
7	70	6	15	338	D3	5.6	14	40	14/95	6/8	32/10	Good
8	52	13	15	70	D10, N20	3.8	7	15	18/95	18/25	52/50	Death
9	52	5	15	300	D3	0.8	12.1	49	10/50	6/8	30/10	Good
10	74	31	15	300	None	4.5	8.64	43	10/50	8/8	32/10	Good

GCS: Glasgow Coma Scale, D: Dopamine, N: Norepinephrine, Bili: Total bilirubin, PLT: Platelet count, and HD: Hemodialysis.

**Table 3:** SOFA, APACHE II, and SAPS II on ICU admission.

Case no.	Age	Sex (M/F)	Admission SOFA	Admission APACHE II	Admission SAPS II	Survived (Y/N)
1	50	M	17	16	50	N
2	56	M	18	17	68	Y
3	52	M	14	16	65	Y
4	22	M	17	13	50	Y
5	58	F	17	18	34	N
6	67	M	9	4	22	Y
7	70	M	14	6	32	Y
8	52	M	18	18	52	N
9	52	M	10	6	30	Y
10	74	M	10	8	32	Y
